# Numerical simulation of 3D Darcy–Forchheimer fluid flow with the energy and mass transfer over an irregular permeable surface

**DOI:** 10.1038/s41598-022-18304-7

**Published:** 2022-08-26

**Authors:** Ebrahem A. Algehyne, Haifaa F. Alrihieli, Anwar Saeed, Fuad S. Alduais, Asif Ullah Hayat, Poom Kumam

**Affiliations:** 1 Department of Mathematics, Faculty of Science, University of Tabuk, P.O.Box 741, Tabuk, 71491 Saudi Arabia; 2 Nanotechnology Research Unit (NRU), University of Tabuk, Tabuk, 71491 Saudi Arabia; 3 Center of Excellence in Theoretical and Computational Science (TaCS-CoE), Faculty of Science, King Mongkut’s University of Technology Thonburi (KMUTT), 126 Pracha Uthit Rd., Bang Mod, Thung Khru, Bangkok, 10140 Thailand; 4 Department of Mathematics, College of Science and Humanities in Al-Aflaj, Prince Sattam Bin Abdulaziz University, Al-Aflaj, 11912 Saudi Arabia; 5 Business Administration Department, Administrative Science College, Thamar University, Thamar, Yemen; 6 Department of Mathematics, Abdul Wali Khan University, Khyber Pakhtunkhwa, Mardan, 23200 Pakistan; 7 Department of Medical Research, China Medical University Hospital, China Medical University, Taichung, 40402 Taiwan

**Keywords:** Engineering, Mathematics and computing

## Abstract

The Jeffrey fluid model is capable of accurately characterizing the stress relaxation behavior of non-Newtonian fluids, which a normal viscous fluid model is unable to perform. The primary objective of this paper is to provide a comprehensive investigation into the effects of MHD and thermal radiation on the 3D Jeffery fluid flow over a permeable irregular stretching surface. The consequences of the Darcy effect, variable thickness and chemical reaction are also considered. The phenomena have been modeled as a nonlinear system of PDEs. Using similarity substitution, the modeled equations are reduced to a dimensionless system of ODEs. The parametric continuation method (PCM) is used to determine the numerical solution to the obtained sets of nonlinear differential equations. The impact of physical parameters on temperature, velocity and mass profiles are presented through Figures and Tables. It has been noticed that the energy profile magnifies with the increment of porosity term, thermal radiation and heat source term, while diminishing with the flourishing upshot of power index and Deborah number. Furthermore, the porosity term and wall thickness parameter enhance the skin friction.

## Introduction

The analysis of heat and mass transmission, as well as boundary layer flow across a starching substrate, is an interesting subject of study due to its numerous applications in various industries and metal extraction processes^1,2^. Sivaraj and Kumar^3^ evaluated the consequences of mixed convection on time-dependent MHD dusty viscous fluid flow with thermal conduction through the impermeable irregular surface. The streamlines of dusty fluids are found to be greater than the velocity distribution of dust particulates. Alharbi et al.^4^ reported the three-dimensional ferrofluid flow across an impermeable upright surface, as well as the impacts of slips in a porous medium with hybrid nanoparticles. It's has been discovered that as the volume proportion of nanoparticles (Nps) increases, the rate of heat transport enhances. Ullah et al.^5^ described the flow variations in the near area of and inside irregular surfaces. Bilal et al.^6^ investigated the effect of inconsistent 2D & 3D sharpness on instability by simulating fully developed flows over two distinct rough substrates. The two designs are created by superimposing sinusoidal functions with various wavelengths and random amplitudes. Gul et al.^7^ and Zhou et al.^8^ established a numerical model that compared the comportment of simple and hybrid NPs moving over an extending sheet. When compared to simple nanofluid, the hybrid nanofluid (HNF) is more efficacious in heat flux due to its excellent thermal properties. Iyyappan and Singh^9^ examined the flow of a force convective laminar boundary layer on an irregular diverging channel when magnetic field influences were employed. Bilal et al.^10^ studied the Casson fluid flow under the upshot of magnetic flux over an expanding surface. It has been shown that the variation of the magnetic field, Marangoni factor, and unsteadiness component decreases the fluid velocity. Some advanced research have been reported by^11–14^.

Non-Newtonian fluids come in a wide variety of types, each with its own set of characteristics. Non-Newtonian fluids are being examined by scientists and researchers due to a broad range of implementations, like drug companies, fiber new tech, cables sealant, food items, crystal growth, psychology and many more. Jeffrey fluid is the most well-known and easiest-to-understand. The Jeffrey fluid parameter and the time retardation parameter elevates this fluid to the top of the non-Newtonian fluids list^15^. Ullah et al.^16^ evaluated the Jeffrey fluid flow across porous horizontal sheet. The velocity of an unsteady Jeffrey fluid flow over an inestimable plane permeable plate is inspected by Algehyne et al.^17^. The results reveal that as the magnetic parameter, the ratio of retardation and relaxation times and Jeffrey fluid parameter increase, the fluid velocity decreases. In the response to an ambient magnetic field, Ali et al.^18^ calculated the upshot of energy conduction on the flow of a Jeffery fluid with immersed NPs through a dynamic flexible substrate. Alrabaiah et al.^19^ inspected the peristaltic transmission of MHD Jeffery fluid flow through channel. Saleem et al. evaluated MHD Jeffrey fluid flows with mass and energy transport on an indefinitely circulating inverted cone.^20^. Azlina et al.^21^ proposed a numerical calculation of the MHD Jeffrey fluid flow through plates in a translucent sheet. Bilal et al.^22^ investigate the 2D Jeffrey fluid flow across a continuously extending disc. Kumar et al.^23^ discussed the influence of an applied magnetics flux on an irregular 2D Jeffrey fluid flow. A theoretical investigation is carried out by Yadav et al.^24^ to determine the upshot of a magnetic flux and mixed convection on the Jeffrey fluid flow. The results show that increasing the Jeffrey fluid parameter reduces system stability while increasing magnetic field parameters has the reverse effect. Recently many researchers have worked on this topic^25–28^.

The MHD flow plays a vital role in manufacturing heavy machinery, astrophysics, electrical power generation solar power equipment, space vehicle and many other fields. Kumar et al.^29^ explore the thermal energy transference in a HNF flow through an extending cylinder while considering magnetic dipoles. Nanoliquid flow across curved stretched sheets is studied numerically by Dhananjaya et al.^30^ to determine the effect of magnetic fields on Casson nanoliquid flow. The findings indicated that enhancing the curvature parameter positively affects the velocity profile, but that it has the opposite impact on the thermal gradient. Chu et al.^31^ scrutinise Maxwell nano liquid’s radiative flow along with a cylinder by taking into consideration the magnetic effect. The fluid flow and temperature fluctuations of nanofluid flow with the Hall upshot are discussed by Acharya et al.^32^. A moving plate with Joule heating is used to demonstrate Magnetohydrodynamic hybrid nanofluid flow with temperature distribution is solved numerically by Lv et al.^33^. Kodi et al.^34^ presented an analytical assessment of Casson fluid flows with heat and mass transmit. This analysis revealed that intensifying the Newtonian heating effect shrinks heat transport at the plate surface. The influence of a porous surface and magnetic flux on the Jeffery fluid flow has been reported by Abdelhameed^35^. Ellahi et al.^36^ investigated the upshots of MHD and velocity slip on sliding flat plate. The obtained outcomes exposed that the velocity contour improves for different values of the slip variable. Recently, a large number of studies have been reported by the implying magnetic effect on the fluid flow^37–40^.

The Jeffrey fluid model effectively describes the stress relaxation behavior of non-Newtonian fluids, which is something that the standard viscous fluid model can't. The Jeffrey fluid model may accurately explain a class of non-Newtonian fluids. The main purpose of this research is to look into the impact of MHD and thermal radiation on the 3D Jeffery fluid flow over an irregular stretching surface. The Darcy effect, varying thickness, and chemical reaction are all taken into account. The results are obtained through computational strategy PCM.

## Mathematical formulation

**Figure 1:**
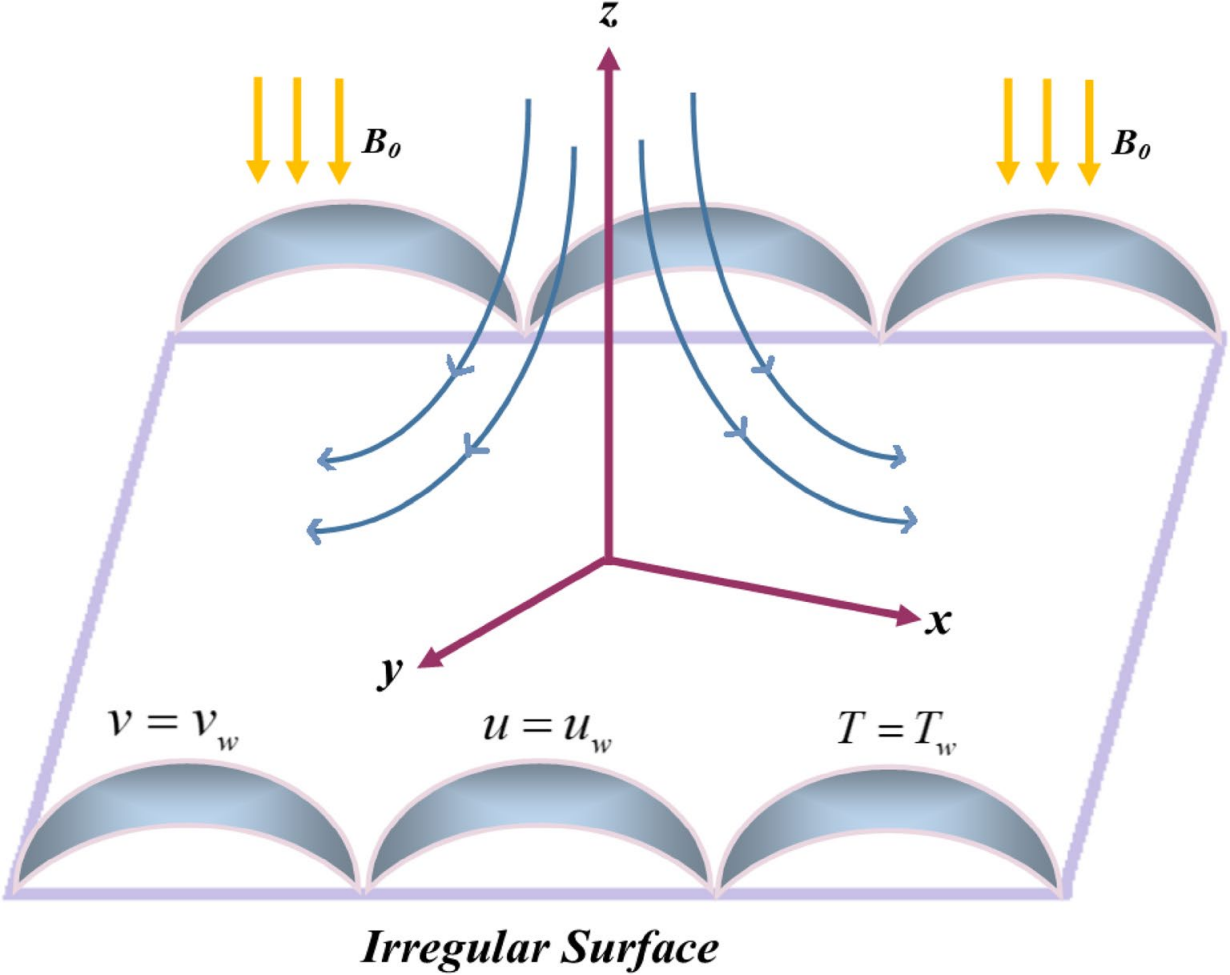
Fluid flow over an irregular permeable surface.

The influence of a tridimensional steady MHD Jeffery fluid flow on an irregular surface immersed in an absorbent medium is considered. Figure 1 described a schematic description of the model. The magnetic effect $B$ is imposed in the *z*-direction. When the fluid is stationary at $t = 0,$ the sheet is impulsively stretched in the *x* and *y* directions with velocities $u_{w}$ and $v_{w}$. The effects of solar radiation on the sheet's surface as well as chemical reaction are considered. Under the above description, the principal equations are expressed as^14^:

1 $$ \frac{\partial u}{{\partial x}} + \frac{\partial v}{{\partial y}} + \frac{\partial w}{{\partial z}} = 0, $$

2 $$ u\frac{\partial u}{{\partial x}} + v\frac{\partial u}{{\partial y}} + w\frac{\partial u}{{\partial z}} = \frac{\nu }{{1 + \Lambda_{1} }}\frac{\partial }{\partial z}\left( {\frac{\partial u}{{\partial z}} + \Lambda_{2} \left( {u\frac{{\partial^{2} u}}{\partial x\partial z} + v\frac{{\partial^{2} u}}{\partial y\partial z} + w\frac{{\partial^{2} u}}{{\partial z^{2} }}} \right)} \right) - \left( {\frac{{\sigma B_{0}^{2} }}{\rho } + \frac{\nu }{{k_{p} }}} \right)u - Fu^{2} , $$

3 $$ u\frac{\partial v}{{\partial x}} + v\frac{\partial v}{{\partial y}} + w\frac{\partial v}{{\partial z}} = \frac{\nu }{{1 + \Lambda_{1} }}\frac{\partial }{\partial z}\left( {\frac{\partial v}{{\partial z}} + \Lambda_{2} \left( {u\frac{{\partial^{2} v}}{\partial x\partial z} + v\frac{{\partial^{2} v}}{\partial y\partial z} + w\frac{{\partial^{2} v}}{{\partial z^{2} }}} \right)} \right) - \left( {\frac{{\sigma B_{0}^{2} }}{\rho } + \frac{\nu }{{k_{p} }}} \right)v - Fv^{2} , $$

4 $$ u\frac{\partial T}{{\partial x}} + v\frac{\partial T}{{\partial y}} + w\frac{\partial T}{{\partial z}} = \left( {\frac{{k_{f} }}{{(\rho C_{p} )_{f} }} + \frac{{16\sigma^{*} T_{\infty }^{3} }}{{3k^{*} \rho C_{p} }}} \right)\frac{{\partial^{2} T}}{{\partial y^{2} }} + \frac{Q}{{\left( {\rho C} \right)_{f} }}\left( {T - T_{\infty } } \right), $$

5 $$ u\frac{\partial C}{{\partial x}} + v\frac{\partial C}{{\partial y}} + w\frac{\partial C}{{\partial z}} = D\frac{{\partial^{2} C}}{{\partial y^{2} }} - k_{1} (C - C_{\infty } ). $$

Here $\left( {u,v,w} \right)$ determine the velocity factors in *x, y* and *z* direction. $k_{p}$ is the permeability of the porous medium, $k_{f}$ is the thermal conductivity, *T* is the temperature of the fluid, $\nu$ is the kinematic viscosity, *Q* the heat absorption/generation term, *F* is the non-uniform inertia factor, where, *C*_*b*_ is the drag coefficient. $D$ is the molecular diffusivity and $\Lambda_{1} ,\Lambda_{2}$ is the period of relaxation and time retardation respectively.

The boundary conditions are^14,41^:

6 $$ \begin{gathered} u - u_{w} (x) = 0,\,\,\,\,v - v_{w} (x) = 0,\,\,\,\,T - T_{w} (x) = 0,\,\,\,\,C - C_{w} (x) = 0\,\,\,{\text{as}}\,\,\,\,z = A\delta^{{\frac{1 - n}{2}}} , \hfill \\ u \to 0,\,\,\,\,v \to 0,\,\,\,\,\,\frac{\partial u}{{\partial z}} \to 0,\,\,\,\,\,\frac{\partial v}{{\partial z}} \to 0,\,\,\,\,T - T_{\infty } ,\,\,C - C_{\infty } \,\,\,\,\,{\text{as}}\,\,\,\,z \to \infty . \hfill \\ \end{gathered} $$

where

$$ \zeta _{1}  = \frac{{k_{B} T}}{{\sqrt 2 \pi d^{2} p}},\quad \Omega  = x + y + z,\quad \zeta _{2}  = \left( {\frac{{2\gamma }}{{\gamma  + 1}}} \right)\frac{{\zeta _{1} }}{{Pr}},\quad B = B_{0} \Omega ^{{\frac{{n - 1}}{2}}} ,u_{w}  = a\Omega ^{{\frac{{n - 1}}{2}}} ,\quad v_{w}  = a{\mkern 1mu} \Omega ^{n} ,\quad T_{w}  - T_{\infty }  = T_{0} \Omega ^{{\frac{{1 - n}}{2}}} \quad n \ne 1. $$

In the above equation, we supposed as $n \ne 1$ (i.e., $n = 1$ denotes the surface shape to flat sheet). Where, $n > 1$ and $n < 1$ are yields to surface curviness, inner convex and outer convex due to reduction and increment of wall thicknesses respectively. $T_{0} ,T_{\infty }$ reference atmospheric liquid temperature $f_{1}$ specify the Maxwell coefficient, $b$ shows the thermal adaptation coefficient, $\zeta_{1} ,\,\zeta_{2}$ are the constant number, $\lambda$ specific heat ratio.

## Similarity transformation

The similarity variables are:

7 $$ \begin{gathered} \zeta = \Omega^{{\frac{n - 1}{2}}} \left( {\frac{{(n + 1)U_{0} }}{{2\nu_{f} }}} \right)^{\frac{1}{2}} z,\,\,\,\psi = \Omega^{{\frac{n + 1}{2}}} \left( {\frac{{2\nu_{f} U_{0} }}{n + 1}} \right)^{\frac{1}{2}} F(\zeta ),\,\,\,\theta (\zeta ) = \frac{{\theta - \theta_{\infty } }}{{\theta_{w} - \theta_{\infty } }},\,\,\phi (\zeta ) = \frac{{\phi - \phi_{\infty } }}{{\phi_{w} - \phi_{\infty } }}, \hfill \\ u = U_{0} \Omega^{\prime \prime}f^{\prime}(\zeta ),\,\,v = V_{0} \Omega^{\prime \prime}g^{\prime}(\zeta ),\,\,w = \left( {\frac{{2\nu_{f} U_{0} }}{n + 1}} \right)^{\frac{1}{2}} \hfill \\ \Omega^{{\frac{n - 1}{2}}} \left[ {\frac{n + 1}{2}\left( {f(\zeta ) + g(\zeta ) + \frac{n - 1}{2}\zeta (f^{\prime}(\zeta ) + g^{\prime}(\zeta ))} \right)} \right], \hfill \\ \end{gathered} $$

By applying the above similarity transformation, Eq. (1) is identically satisfied while Eq. (2–5) take the form as:

8 $$ f^{\prime \prime \prime} + (\Lambda_{1} + 1)\left( {\left( {g + f} \right)f^{\prime \prime} - \frac{2n}{{n + 1}}\left( {Frf^{{\prime}{2}} + f^{\prime}g^{\prime}} \right)} \right) + D\left( {\left( {\frac{3n - 1}{2}} \right)\left( {g^{\prime \prime} + f^{\prime \prime}} \right)f^{\prime \prime} + \left( {n - 1} \right)\left( {f^{\prime} + g^{\prime}} \right)f^{\prime \prime \prime}} \right) - D\left( {\frac{n + 1}{2}(g + f)f^{\prime \prime \prime} + \frac{2}{n + 1}(1 + \Lambda_{1} )(M_{F}^{2} + P_{0} )f^{\prime}} \right) = 0, $$

9 $$ g^{\prime \prime \prime} + (1 + \Lambda_{1} )\left( {(g + f)g^{\prime \prime} - \frac{2n}{{n + 1}}(Frg^{{\prime}{2}} + g^{\prime}\,f^{\prime})} \right) + D\left( {\left( {\frac{3n - 1}{2}} \right)(g^{\prime \prime} + f^{\prime \prime})g^{\prime \prime} + (n - 1)(f^{\prime} + g^{\prime})g^{\prime \prime \prime}} \right) - D\left( {\frac{n + 1}{2}(g + f)g^{\prime \prime \prime} + \frac{2}{n + 1}(1 + \Lambda_{1} )(M_{F}^{2} + P_{0} )g^{\prime}} \right) = 0, $$

10 $$ (1 + R)\theta^{\prime \prime} + (f + g)Pr\theta^{\prime} + Hs\theta = 0, $$

11 $$ \phi^{\prime \prime} + Sc\left( {(f + g)\phi^{\prime} - \frac{2}{n + 1}C_{r} \phi } \right) = 0. $$

The reduced conditions are:

12 $$ \left. \begin{aligned} g(\eta ) & = f(\eta ) = \Lambda \left( {\frac{1 - n}{{1 + n}}} \right),\quad f^{\prime}(\eta ) = \phi (\eta ) = g^{\prime}(\eta ) = \theta (\eta ) = 1\;{\text{as}}\;\eta \to 0 \\ f^{\prime}(\eta ) & = g^{\prime}(\eta ) \to 0,\quad f^{\prime \prime}(\eta ) = g^{\prime \prime}(\eta ) = \phi^{\prime}(\eta ) = \theta^{\prime}(\eta ) \to 0\;{\text{as}}\;\eta \to \infty . \\ \end{aligned} \right\} $$

Here, *D* and $M_{F}$ is the Deborah number and magnetic field, *Hs* is the absorption & generation term, $P_{0}$ is the porosity factor, *R* is the thermal radiation, *Pr* and *Sc* is the Prandtl and Schmidt numbers, *Fr* is the Darcy Forchhemier term, $C_{r}$ is the chemical reaction and $\Lambda$ wall thickness factor. Mathematically we have

13 $$ \left. \begin{gathered} P_{0} = \frac{{v_{f} }}{{k_{p} U_{0} \Omega^{n - 1} }},\,\,\,Pr = \frac{{\mu_{f} (C_{p} )_{f} }}{{k_{f} }},\,\,\,\Lambda = z\left( {\frac{{(n + 1)U_{0} }}{2\nu }} \right)^{\frac{1}{2}} ,\,\,M_{F} = \frac{{\sigma B_{0}^{2} }}{{\rho U_{0} \Omega^{n - 1} }}, \hfill \\ Hs = \frac{xQ}{{u\rho C_{p} }},\,\,\,\,D = \Lambda_{2} U_{0} \Omega^{n - 1} ,\,\,\,\,\,Fr = \frac{{xC_{b} }}{{k^{*1/2} }},\,\,\,\,\,C_{r} = \frac{{k_{1} }}{{U_{0} \Omega^{n - 1} }},\,\,\,\,\,Sc = \frac{{\nu_{f} }}{D}. \hfill \\ \end{gathered} \right\} $$

The friction factor towards *x* and *y* direction are:

14 $$ Cf_{x} = \frac{{\tau_{xz} }}{{\rho U_{w}^{2} }},\,\,\,Cf_{y} = \frac{{\tau_{yz} }}{{\rho U_{w}^{2} }}, $$

where

15 $$ \begin{gathered} \tau_{xz} = \frac{\mu }{{1 + \Lambda_{1} }}\left[ {\frac{\partial u}{{\partial z}} + \left( {u\frac{{\partial^{2} u}}{\partial x\partial z} + v\frac{{\partial^{2} u}}{\partial y\partial z} + w\frac{{\partial^{2} u}}{{\partial z^{2} }}} \right)\Lambda_{2} } \right], \hfill \\ \tau_{yz} = \frac{\mu }{{1 + \Lambda_{1} }}\left[ {\frac{\partial v}{{\partial z}} + \left( {u\frac{{\partial^{2} v}}{\partial x\partial z} + v\frac{{\partial^{2} v}}{\partial y\partial z} + w\frac{{\partial^{2} v}}{{\partial z^{2} }}} \right)\Lambda_{2} } \right]. \hfill \\ \end{gathered} $$

Here the physical quantities are:

16 $$ Nu_{x} = \frac{{\Omega q_{w} }}{{k_{f} (T_{w} - T_{\infty } )}},\quad {\text{where}}\quad q_{w} = - \left( {k_{f} + \frac{{16\sigma^{*} T_{\infty }^{3} }}{{3k^{*} }}} \right)\frac{\partial T}{{\partial z}}. $$

17 $$ Sh_{x} = \frac{{\Omega j_{w} }}{{D(C_{w} - C_{\infty } )}},\quad {\text{where}}\quad j_{w} = - D\frac{\partial C}{{\partial z}}, $$

The skin friction, heat and mass allocation expression are as follows:

18 $$ {\text{Re}}_{x}^{0.5} Cf_{x} = \frac{1}{{1 + \Lambda_{1} }}\left( {\frac{n + 1}{2}} \right)^{0.5} \left( {f^{\prime \prime}(0) + D\left( {\frac{3n - 1}{2}f^{\prime \prime}(0)(f^{\prime}(0) + g^{\prime}(0)) - \frac{n + 1}{2}f^{\prime \prime \prime}(0)(g(0) + f(0))} \right)} \right), $$

19 $$ {\text{Re}}_{y}^{0.5} Cf_{y} = \frac{1}{{1 + \Lambda_{1} }}\left( {\frac{n + 1}{2}} \right)^{0.5} \left( {g^{\prime \prime}(0) + D\left( {\frac{3n - 1}{2}g^{\prime \prime}(0)(f^{\prime}(0) + g^{\prime}(0)) - \frac{n + 1}{2}(g(0) + f(0))g^{\prime \prime \prime}(0)} \right)} \right), $$

20 $$ Nn_{x} {\text{Re}}_{{}}^{\frac{1}{2}} = - \left( {\frac{n + 1}{2}} \right)\theta^{\prime}(0),\,\,\,\,\,\,\,\,Sh_{x} {\text{Re}}_{{}}^{{\frac{ - 1}{2}}} = - \left( {\frac{n + 1}{2}} \right)\phi^{\prime}(0).\, $$

Here ${\text{Re}} = \frac{{\Omega U_{w} }}{{\nu_{f} }}$ is the Reynold’s number.

## Numerical solution

The basic methodology steps of PCM approach are as follow^17,42–46^:

### Step 1: simplification to 1st order ODE

21 $$ \left. \begin{aligned} \hbar_{1} & = f(\eta ),\;\hbar_{3} = f^{\prime\prime}(\eta ),\;\hbar_{5} = g^{\prime}(\eta ),\;\hbar_{7} = \theta (\eta ),\;\hbar_{9} = \phi (\eta ), \\ \hbar_{2} & = f^{\prime}(\eta ),\;\hbar_{4} = g(\eta ),\;\hbar_{6} = g^{\prime \prime}(\eta ),\;\hbar_{8} = \theta^{\prime}(\eta ),\;\hbar_{10} = \phi^{\prime}(\eta ). \\ \end{aligned} \right\} $$

By putting Eq. (21) in Eqs. (12)–(15) & (16), we get:

22 $$ \left( {1 - D\frac{{n + 1}}{2}(\hbar _{1}  + \hbar _{4} ) + D(n - 1)(\hbar _{2}  + \hbar _{5} )} \right)\hbar _{3}^{\prime }  + (1 + \Lambda _{1} )\left( {(\hbar _{1}  + \hbar _{4} )\hbar _{3}  - \frac{{2n}}{{n + 1}}(Fr\hbar _{2}^{2}  + \hbar _{2} \hbar _{5} )} \right)\quad  + D\frac{{3n - 1}}{2}(\hbar _{3}  + \hbar _{6} )\hbar _{3}  - \frac{{2D}}{{n + 1}}(1 + \Lambda _{1} )(M_{F}^{2}  + P_{0} )\hbar _{2}  = 0, $$

23 $$ \left( {1 - D\frac{{n + 1}}{2}(\hbar _{1}  + \hbar _{4} ) + D(n - 1)(\hbar _{2}  + \hbar _{5} )} \right)\hbar _{6}^{\prime }  + (1 + \Lambda _{1} )\left( {(\hbar _{4}  + \hbar _{5} )\hbar _{6}  - \frac{{2n}}{{n + 1}}(Fr\hbar _{5}^{2}  + \hbar _{2} \hbar _{5} )} \right)\quad  + D\frac{{3n - 1}}{2}(\hbar _{2}  + \hbar _{6} )\hbar _{6}  - D\frac{2}{{n + 1}}(1 + \Lambda _{1} )(M_{F}^{2}  + P_{0} )\hbar _{5}  = 0, $$

24 $$ (1 + R)\hbar^{\prime}_{8} + (\hbar_{1} + \hbar_{4} )Pr\hbar_{8} + Hs\hbar_{7} = 0, $$

25 $$ \hbar^{\prime}_{10} + Sc\left( {(\hbar_{1} + \hbar_{4} )\hbar_{10} - \frac{2}{n + 1}C_{r} \hbar_{9} } \right) = 0. $$

with the corresponding boundary conditions.

26 $$ \left. \begin{gathered} \hbar_{1} (\eta ) = \hbar_{4} (\eta ) = \Lambda \left( {\frac{1 - n}{{1 + n}}} \right),\,\,\,\hbar_{2} (\eta ) = \,\hbar_{5} (\eta ) = \hbar_{7} (\eta ) = \hbar_{9} (\eta ) = 1\,\,\,\,{\text{as}}\,\,\,\,\,\eta \to 0 \hfill \\ \hbar_{2} (\eta ) = \hbar_{5} (\eta ) = 0,\,\,\,\,\,\hbar_{3} (\eta ) = \hbar_{6} (\eta ) = \hbar_{8} (\eta ) = \hbar_{10} (\eta ) = 0\,\,\,\,\,{\text{as}}\,\,\,\,\,\eta \to \infty . \hfill \\ \end{gathered} \right\} $$

### Step 2: familiarizing the embedding constraint *p* in Eqs. (22)–(25)

27 $$ \begin{gathered} \left( {1 - D\frac{n + 1}{2}(\hbar_{1} + \hbar_{4} ) + D(n - 1)(\hbar_{2} + \hbar_{5} )} \right)\hbar^{\prime}_{3} + \left( {(1 + \Lambda_{1} )(\hbar_{1} + \hbar_{4} ) + D\frac{3n - 1}{2}(\hbar_{3} + \hbar_{6} )} \right)(\hbar_{3} - 1)p \hfill \\ - \frac{{2n(1 + \Lambda_{1} )}}{n + 1}(Fr\hbar_{2}^{2} + \hbar_{2} \hbar_{5} ) - \frac{2D}{{n + 1}}(1 + \Lambda_{1} )(M_{F}^{2} + P_{0} )\hbar_{2} = 0, \hfill \\ \end{gathered} $$

28 $$ \begin{gathered} \left( {1 - D\frac{n + 1}{2}(\hbar_{1} + \hbar_{4} ) + D(n - 1)(\hbar_{2} + \hbar_{5} )} \right)\hbar^{\prime}_{6} + \left( {(1 + \Lambda_{1} )(\hbar_{4} + \hbar_{5} ) + D\frac{3n - 1}{2}(\hbar_{2} + \hbar_{6} )} \right)(\hbar_{6} - 1)p \hfill \\ - \frac{{2n(1 + \Lambda_{1} )}}{n + 1}(Fr\hbar_{5}^{2} + \hbar_{2} \hbar_{5} ) - D\frac{2}{n + 1}(1 + \Lambda_{1} )(M_{F}^{2} + P_{0} )\hbar_{5} = 0, \hfill \\ \end{gathered} $$

29 $$ (1 + R)\hbar^{\prime}_{8} + (\hbar_{1} + \hbar_{4} )Pr(\hbar_{8} - 1)p + Hs\hbar_{7} = 0, $$

30 $$ \hbar^{\prime}_{10} + Sc\left( {(\hbar_{1} + \hbar_{4} )(\hbar_{10} - 1)p - \frac{2}{n + 1}C_{r} \hbar_{9} } \right) = 0. $$

### Step 3: solving the Cauchy principal

Numerical implicit scheme is employed for the above modeled equations, which is defined as below:

31 $$ \frac{{U^{i + 1} - U^{i} }}{\Delta \eta } = \rlap{---} \Delta U^{i + 1} ,\,\,\,\,\frac{{W^{i + 1} - W^{i} }}{\Delta \eta } = \rlap{---} \Delta W^{i + 1} . $$

Finally, we get:

32 $$ U^{i + 1} = (I - \Delta \rlap{---} \Delta \eta )^{ - 1} U^{i} ,\,\,\,\,\,\,\,\,W^{i + 1} = (I - \Delta \rlap{---} \Delta \eta )^{ - 1} (W^{i} + \Delta \eta R). $$

## Results and discussion

The section revealed the physics behind each figure and table. The subsequent trends have been observed:

### Velocity profile

**Figure 2:**
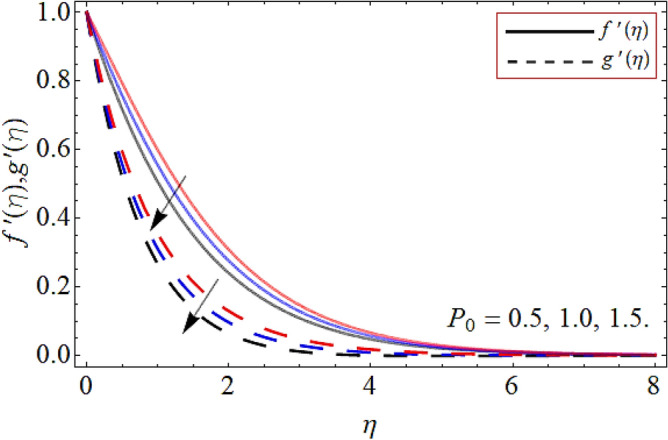
Velocity profile versus porosity term *P*_*0*_.

**Figure 3:**
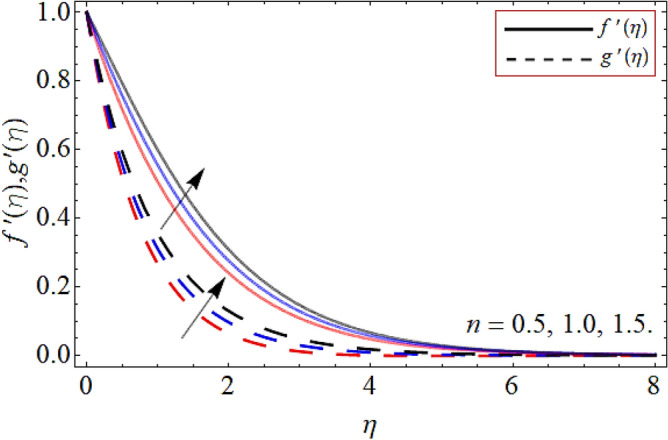
Velocity profile versus power law index *n*.

**Figure 4:**
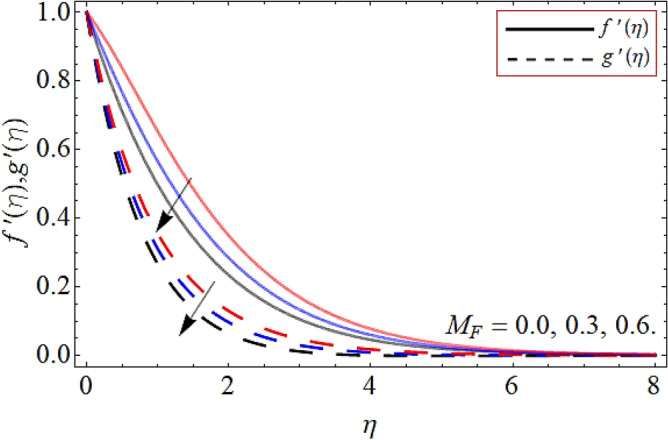
Velocity profile versus magnetic field constraint *M*_*F*._

**Figure 5:**
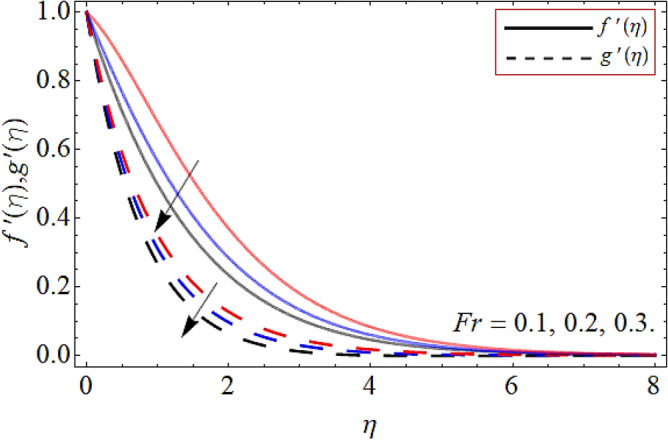
Velocity outline versus Darcy Forchhemier term *Fr*.

**Figure 6:**
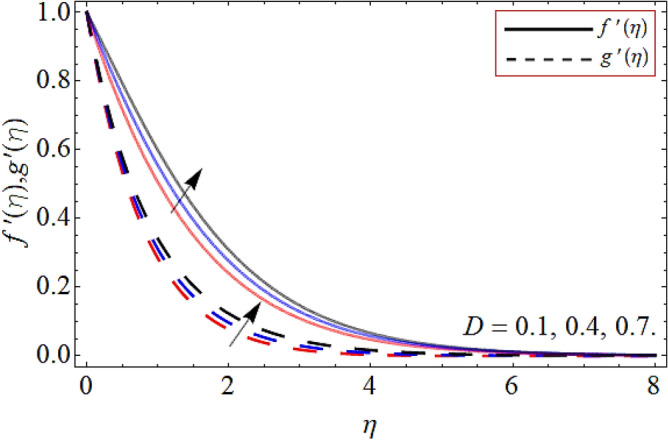
Velocity outline versus local Deborah number *D*.

**Figure 7:**
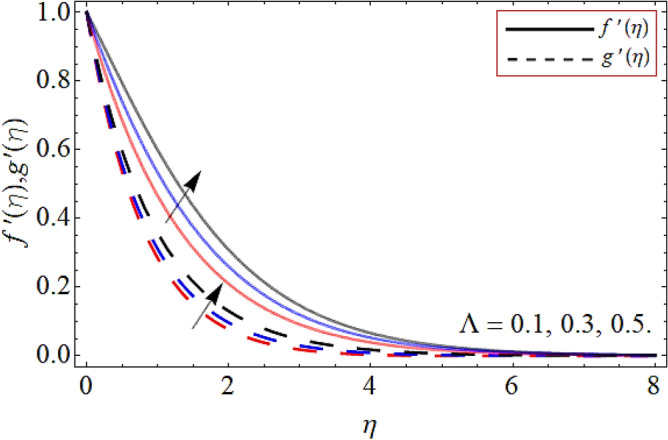
Velocity profile versus wall thickness term $\Lambda$.

**Figure 8:**
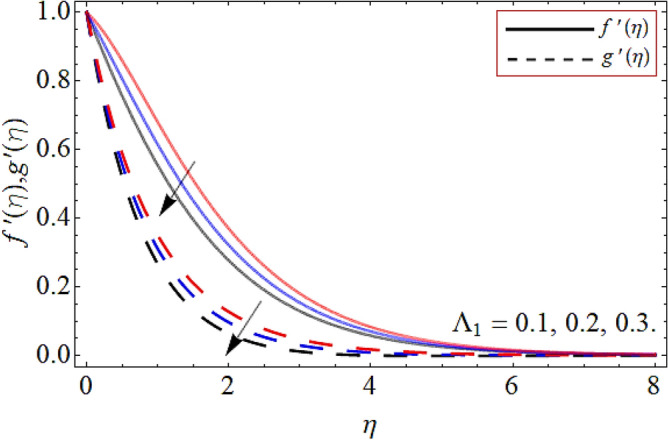
Velocity profile versus ratio of relaxation time to retardation term $\Lambda_{1}$.

Figures 2, 3, 4, 5, 6, 7, 8 explained the presentation of axial $f^{\prime}\left( \eta \right)$ and radial $g^{\prime}\left( \eta \right)$ velocity profile versus the variation of porosity term *P*_*0*_, power-law index *n*, magnetic field constraint *M*_*F*_*,* Darcy Forchhemier term *Fr*, Deborah number *D*, wall thickness term $\Lambda$ and the ratio of relaxation time to retardation term $\Lambda_{1}$ respectively. Figures 2 & 3 shows that the velocity curve declines with the growth of porosity term, while augmented with the flourishing upshot of power index constraint. Physically, the number of pours increases with the porosity parameter effect, which resists the fluid flow, so causes the reduction in the velocity outline. Figures 4 and 5 reported that the rising frequency of both constraints magnetic field and Darcy Forchhemier effect deduce the velocity distribution. Because the opposing force, which is created due to magnetic effect, resist the flow field, as a result such trend observed. Figures 6 and 7 described that the impact of local Deborah number and wall thickness parameter augmented the velocity field. Figure 8 displays that the upshot of thermal relaxation term, reduce the energy profile.

### Energy profile

**Figure 9:**
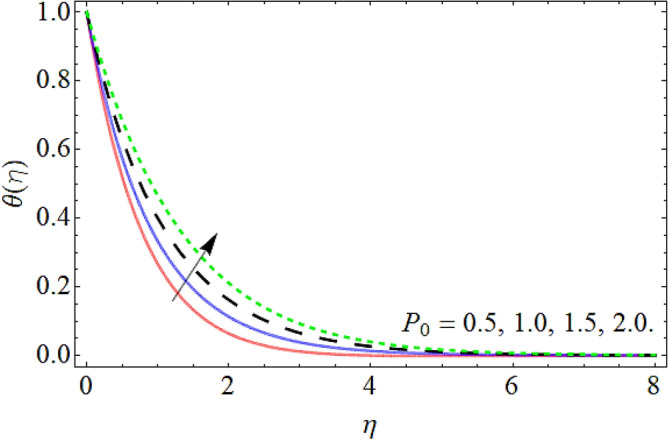
Energy profile versus the porosity term *P*_*0*_.

**Figure 10:**
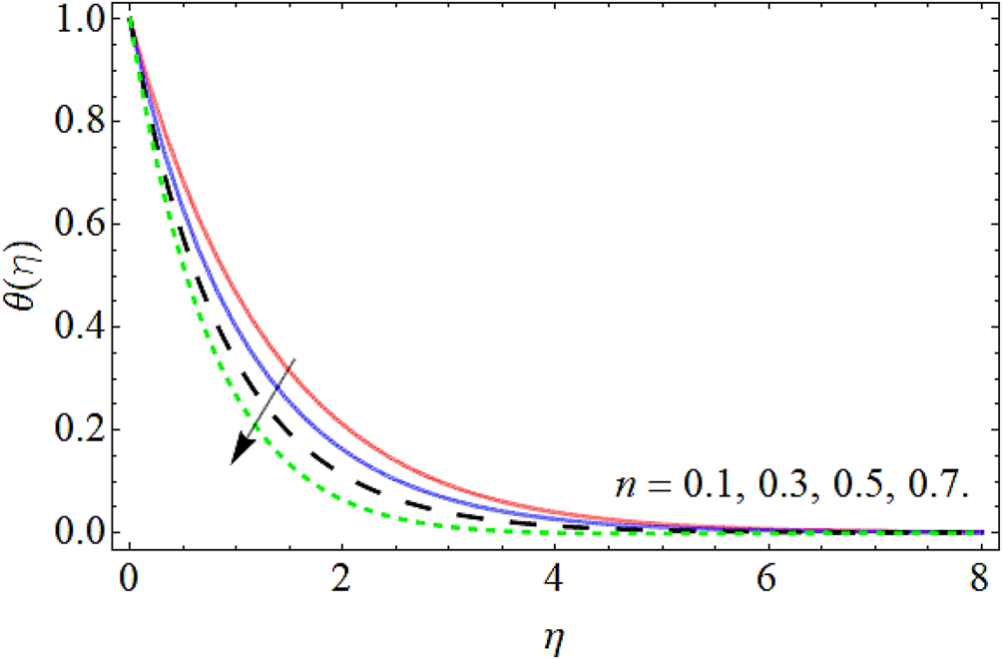
Energy profile versus the power law index *n*.

**Figure 11:**
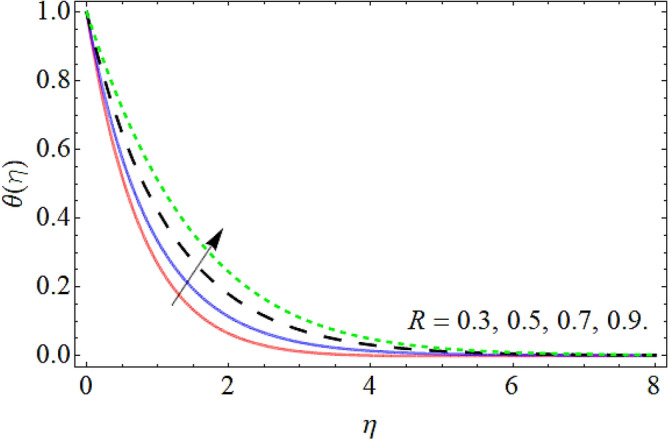
Energy profile versus the thermal radiation *R*.

**Figure 12:**
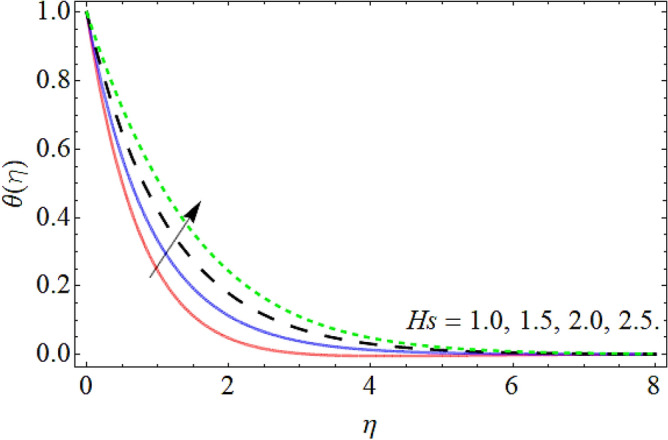
Energy profile versus the heat source term *Hs*.

**Figure 13:**
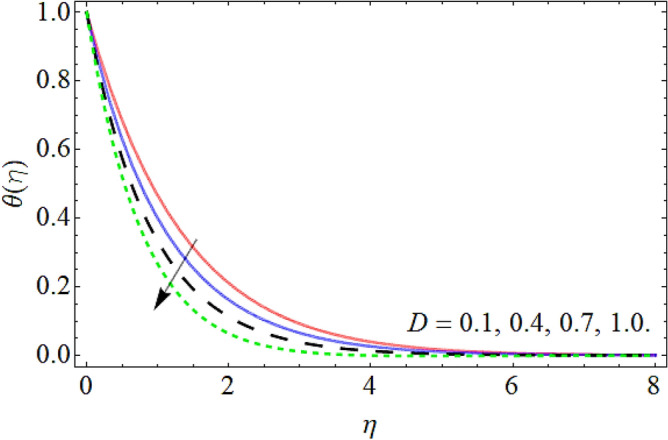
Energy profile versus the local Deborah number *D*.

**Figure 14:**
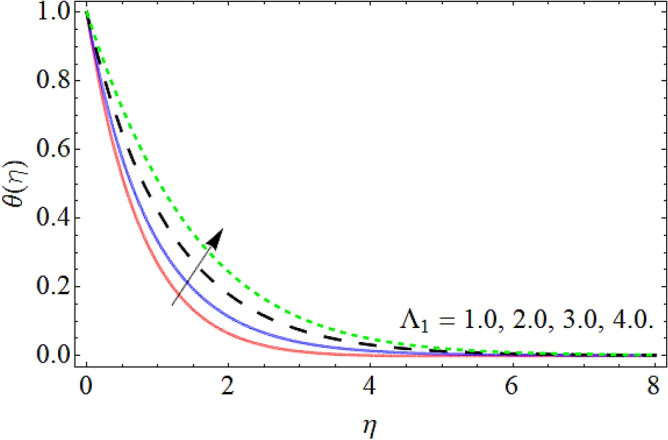
Energy profile versus the ratio of relaxation time to retardation term $\Lambda_{1}$.

Figures 9, 10, 11, 12, 13, 14 explained the appearance of energy contour $\theta \left( \eta \right)$ versus the variation of porosity term *P*_*0*_, power-law index *n*, thermal radiation *R*, heat source term *Hs*, Deborah number *D*, thermal relaxation term $\Lambda_{1}$. Figures 9 and 10 shows that the energy contour amplifies with the increment of porosity term while diminishing with the flourishing upshot of power index constraint. Physically, the number of pours increases with the porosity parameter effect, which resists the fluid flow, so triggers an expansion in the heat. Figures 11 and 12 illustrated that the thermal field boosts with the mounting values of *R* and *Hs*. The effect of both constraints generates an additional heat inside the fluid, which scores in the advancement of the temperature field $\theta \left( \eta \right)$. Figures 13 and 14 presented that Deborah number *D* decreases the thermal energy field, while the impact of thermal relaxation term $\Lambda_{1}$ enhances the energy distribution.

### Concentration profile

**Figure 15:**
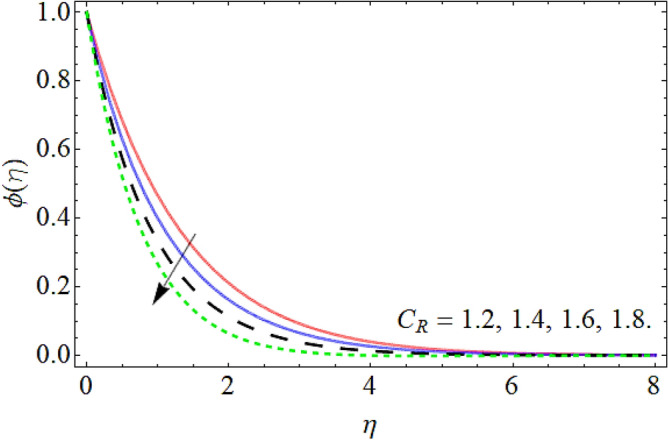
Mass profile versus the chemical reaction *C*_*R*_.

**Figure 16:**
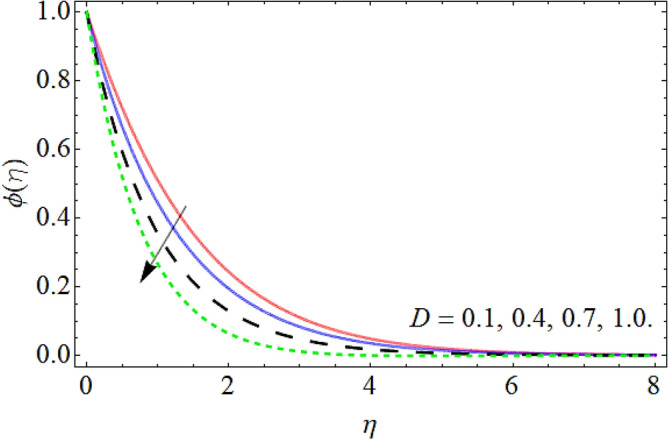
Mass profile versus the Deborah number *D*.

**Figure 17:**
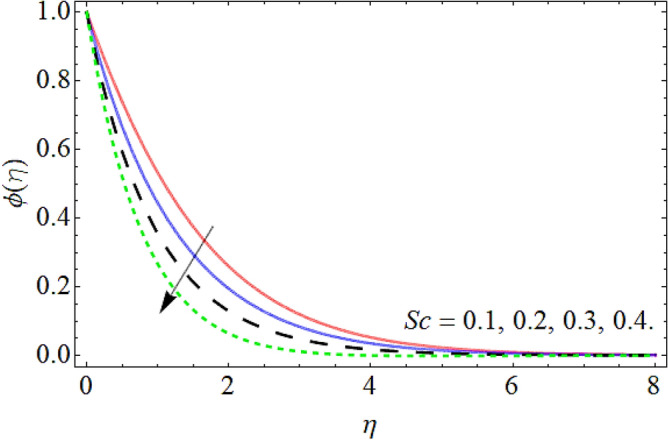
Mass profile versus the Schmidt number *Sc*.

Figures 15, 16 and 17 elaborated the exhibition of mass outline $\phi \left( \eta \right)$ versus *C*_*R*_, Deborah number *D* and Schmidt number *Sc* respectively. Figures 15, 16 and 17 assessed hat the mass transmission profiles reduce with the intensifying upshot of chemical reaction, Deborah number and Schmidt number. Physically, the influence of the *Sc* enhances the kinetic viscosity of the fluid, while lessen the molecular diffusion, which causes the reduction in mass profile. Similarly, the consequences of chemical reaction and Deborah number also declines the concentration profile $\phi \left( \eta \right).$

### Error analysis

**Figure 18:**
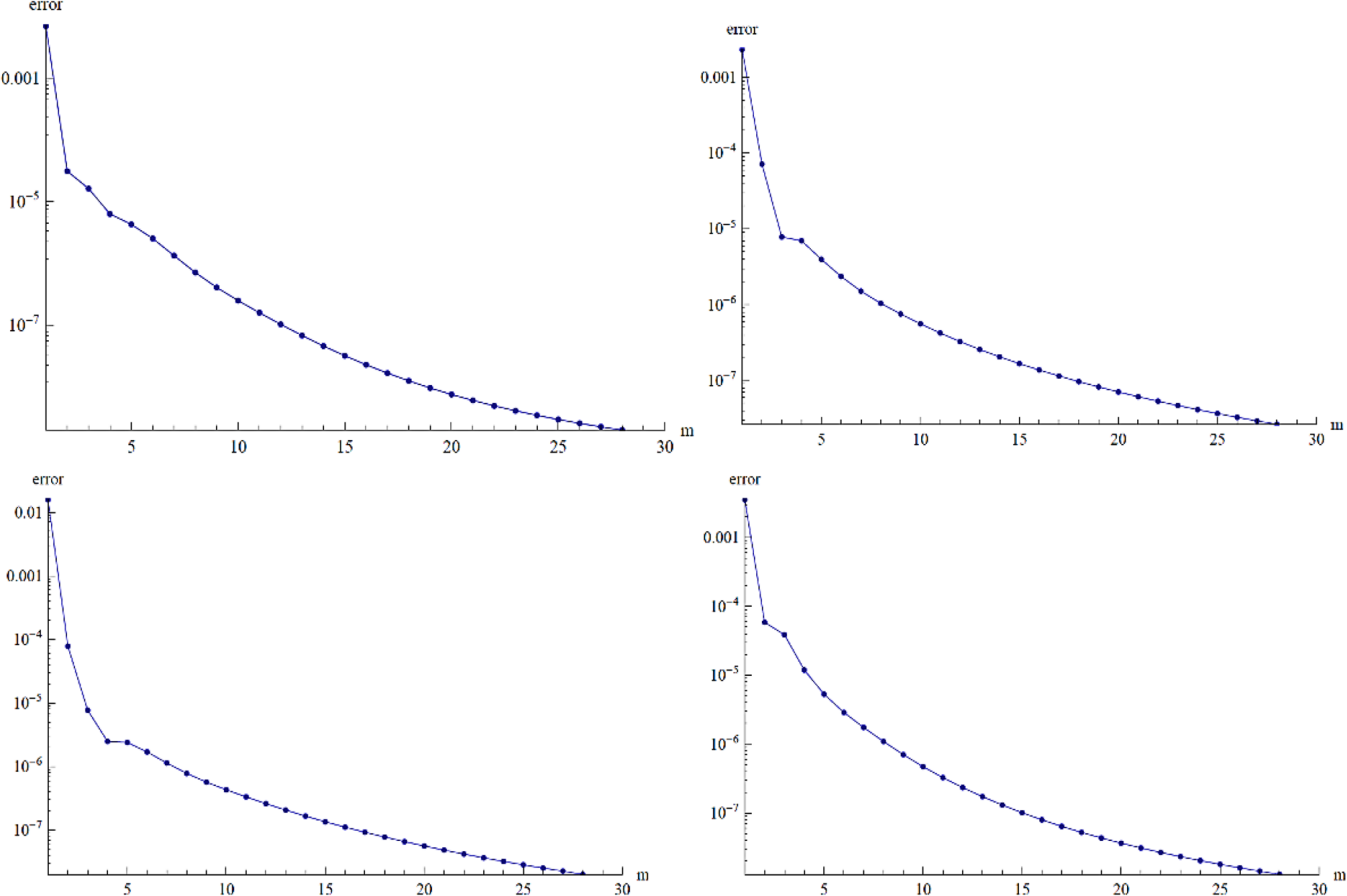
Residual error for velocity, energy and concentration profile.

In Fig. 18, we performed the error analysis, to ensure that our results are accurate up to the lowest residual error scale. Until evaluating and providing physical forecasts, we analyze an error to determine the accuracy of the proposed method.

Tables 1 and 2 illustrated the statistical outcomes for skin friction, Nusselt and Sherwood number versus several physical constraints respectively. Table 3 highlighted the comparative assessment of the present results versus the existing works. The results of Table 3 verify the accuracy of the current analysis.

**Table 1:** The arithmetical results for skin friction along *x* and *y* direction.

$$\Lambda_{1}$$	$$\Lambda$$	$$M$$	$$P_{0}$$	$$D$$	$$Cf_{x} Re_{x}^{{{\raise0.7ex\hbox{$1$} \!\mathord{\left/ {\vphantom {1 2}}\right.\kern-\nulldelimiterspace} \!\lower0.7ex\hbox{$2$}}}}$$	$$Cf_{y} Re_{y}^{{{\raise0.7ex\hbox{$1$} \!\mathord{\left/ {\vphantom {1 2}}\right.\kern-\nulldelimiterspace} \!\lower0.7ex\hbox{$2$}}}}$$
0.3	0.1	0.4	1.0	0.2	− 1.47496	− 2.63760
0.5					− 1.38337	− 2.31511
0.7					− 1.30327	− 2.03290
	0.2				− 1.53371	− 2.48535
	0.3				− 1.59228	− 2.39394
		0.8			− 1.72496	− 3.04651
		1.2			− 1.87555	− 3.41097
			1.5		− 1.64601	− 3.02635
			2.0		− 1.79842	− 3.04401
				0.4	− 1.49881	− 3.08712
				0.6	− 1.52651	− 3.53664

**Table 2:** The statistical outputs of Sherwood and Nusselt numbers.

$\Lambda_{1}$	$\Lambda$	$M$	$C_{R}$	$D$	$\left( { - \frac{n + 1}{2}} \right)\theta^{\prime}\left( 0 \right)$	$\left( { - \frac{n + 1}{2}} \right)\phi^{\prime}\left( 0 \right)$
0.1	1.0	0.4	1.0	0.2	0.5807	0.8134
0.3					0.5730	0.8117
0.5					0.5657	0.8102
	2.0				0.1523	0.8133
	3.0				0.9234	0.8133
		0.8			0.5552	0.8078
		1.2			0.5225	0.2013
			1.5		0.5807	1.2574
			2.0		0.5807	1.2814
				0.4	0.6013	0.9963
				0.4	0.6177	0.9891

**Table 3:** Relative evaluation of current results with the available literature for $- f^{\prime \prime}(0)$.

*n*	Reddy et al.^41^	Khader et al.^47^	Khan et al.^48^	Present work
0.0	0.95664	0.9477	0.94128	0.95136
0.5	0.97894	0.9698	0.97354	0.97365
0.0	1.01000	1.0100	0.98364	0.99372
2.0	1.02242	1.0134	1.01270	1.02281
3.0	1.03488	1.0258	1.02454	1.03464
5.0	1.04762	1.0386	1.03758	1.04767
10.0	1.06134	1.0503	1.04994	1.16995

## Conclusion

We have numerically analyzed the energy conveyance through Jeffery fluid flow over an irregular extensible sheet with a porous medium. The consequences of the Darcy effect, variable thickness and chemical reaction are also considered. The phenomena have been modeled as a system of PDEs. Using similarity substitution, the modeled equations are reduced to a dimensionless system of ODEs. The computational technique is used to determine the numerical solution to the obtained sets of nonlinear differential equations. The key conclusions are:

- The velocity profiles $\left( {f^{\prime}\left( \eta \right),\,\,g^{\prime}\left( \eta \right)} \right)$ both decline with the increment of porosity term, magnetic field, Darcy Forchhemier and thermal relaxation factor while augmented with the flourishing upshot of power index and local Deborah number.
- The energy profile $\theta \left( \eta \right)$ magnifies with the increment of porosity term, thermal radiation and heat source term, while diminishing with the flourishing upshot of power index and Deborah number.
- The mass transfer profiles reduce with the rising upshot of *C*_*R*_, Deborah number and Schmidt number.
- The porosity term and wall thickness parameter enhance the skin friction.

## Data Availability

All data used in this manuscript have been presented within the article.

## References

[1] Elattar S, Helmi MM, Elkotb MA, El-Shorbagy MA, Abdelrahman A, Bilal M, Ali A (2022). Computational assessment of hybrid nanofluid flow with the influence of hall current and chemical reaction over a slender stretching surface. Alex. Eng. J..

[2] Bilal M, Saeed A, Selim MM, Gul T, Ali I, Kumam P (2021). Comparative numerical analysis of Maxwell's time-dependent thermo-diffusive flow through a stretching cylinder. Case Stud. Therm. Eng..

[3] Sivaraj R, Kumar BR (2013). Chemically reacting dusty viscoelastic fluid flow in an irregular channel with convective boundary. Ain Shams Eng. J..

[4] Alharbi KAM, Ahmed AES, Ould Sidi M, Ahammad NA, Mohamed A, El-Shorbagy MA, Marzouki R (2022). Computational valuation of darcy ternary-hybrid nanofluid flow across an extending cylinder with induction effects. Micromachines.

[5] Ullah I, Ullah R, Alqarni MS, Xia WF, Muhammad T (2021). Combined heat source and zero mass flux features on magnetized nanofluid flow by radial disk with the applications of Coriolis force and activation energy. Int. Commun. Heat Mass Transfer.

[6] Bilal M, Saeed A, Gul T, Kumam W, Mukhtar S, Kumam P (2022). Parametric simulation of micropolar fluid with thermal radiation across a porous stretching surface. Sci. Rep..

[7] Gul T, Khan A, Bilal M, Alreshidi NA, Mukhtar S, Shah Z, Kumam P (2020). Magnetic dipole impact on the hybrid nanofluid flow over an extending surface. Sci. Rep..

[8] Zhou SS, Bilal M, Khan MA, Muhammad T (2021). Numerical analysis of thermal radiative maxwell nanofluid flow over-stretching porous rotating disk. Micromachines.

[9] Iyyappan G, Singh AK (2021). MHD flows on irregular boundary over a diverging channel with viscous dissipation effect. Int. J. Numer. Meth. Heat Fluid Flow.

[10] Bilal M, Saeed A, Gul T, Rehman M, \& Khan, A. (2021). Thin-film flow of Carreau fluid over a stretching surface including the couple stress and uniform magnetic field. Partial Differ. Equ. Appl. Math..

[11] Ahmed N, Khan U, Mohyud-Din ST, Erturk VS (2017). Influence of thermal and concentration gradients on unsteady flow over a stretchable surface. Results Phys..

[12] Khan M, Rasheed A (2022). Numerical implementation and error analysis of nonlinear coupled fractional viscoelastic fluid model with variable heat flux. Ain Shams Eng. J..

[13] Ahmadian A, Bilal M, Khan MA, Asjad MI (2020). Numerical analysis of thermal conductive hybrid nanofluid flow over the surface of a wavy spinning disk. Sci. Rep..

[14] Khan M, Rasheed A (2021). Slip velocity and temperature jump effects on molybdenum disulfide MoS2 and silicon oxide SiO2 hybrid nanofluid near irregular 3D surface. Alex. Eng. J..

[15] Ahmed N, Khan U, Mohyud-Din ST (2018). A theoretical investigation of unsteady thermally stratified flow of γAl2O3− H2O and γAl2O3− C2H6O2 nanofluids through a thin slit. J. Phys. Chem. Solids.

[16] Ullah Z, Ullah I, Zaman G, Khan H, Muhammad T (2021). Mathematical modeling and thermodynamics of Prandtl-Eyring fluid with radiation effect: a numerical approach. Sci. Rep..

[17] Algehyne EA, Areshi M, Saeed A, Bilal M, Kumam W, Kumam P (2022). Numerical simulation of bioconvective Darcy Forchhemier nanofluid flow with energy transition over a permeable vertical plate. Sci. Rep..

[18] Ali A, Maqsood M, Anjum HJ, Awais M, Sulaiman M (2022). Analysis of heat transfer on MHD Jeffrey nanofluid flow over nonlinear elongating surface of variable thickness. ZAMM J. Appl. Math. Mechanics.

[19] Alrabaiah H, Bilal M, Khan MA, Muhammad T, Legas EY (2022). Parametric estimation of gyrotactic microorganism hybrid nanofluid flow between the conical gap of spinning disk-cone apparatus. Sci. Rep..

[20] Saleem S, Al-Qarni MM, Nadeem S, Sandeep N (2018). Convective heat and mass transfer in magneto Jeffrey fluid flow on a rotating cone with heat source and chemical reaction. Commun. Theor. Phys..

[21] Noor NAM, Shafie S, Admon MA (2020). Unsteady MHD squeezing flow of Jeffrey fluid in a porous medium with thermal radiation, heat generation/absorption and chemical reaction. Phys. Scr..

[22] Bilal M, Ahmed AES, El-Nabulsi RA, Ahammad NA, Alharbi KAM, Elkotb MA, SA, Z. A. (2022). Numerical analysis of an unsteady, electroviscous, ternary hybrid nanofluid flow with chemical reaction and activation energy across parallel plates. Micromachines.

[23] Ojjela O, Raju A, Kumar NN (2019). Influence of induced magnetic field and radiation on free convective Jeffrey fluid flow between two parallel porous plates with Soret and Dufour effects. J. Mech..

[24] Yadav D, Mohamad AA, Awasthi MK (2021). The Horton–Rogers–Lapwood problem in a Jeffrey fluid influenced by a vertical magnetic field. Proc. Inst. Mech. Eng. Part E J. Process Mech. Eng..

[25] Khan M, Lone SA, Rasheed A, Alam MN (2022). Computational simulation of Scott-Blair model to fractional hybrid nanofluid with Darcy medium. Int. Commun. Heat Mass Transfer.

[26] Khan U, Ahmed N, Mohyud-Din ST, Alsulami MD, Khan I (2022). A novel analysis of heat transfer in the nanofluid composed by nanodimaond and silver nanomaterials: numerical investigation. Sci. Rep..

[27] Khan M, Rasheed A (2021). The space–time coupled fractional Cattaneo-Friedrich Maxwell model with Caputo derivatives. Int. J. Appl. Comput. Math..

[28] Ali A, Awais M, Al-Zubaidi A, Saleem S, Marwat DK (2022). Hartmann boundary layer in peristaltic flow for viscoelastic fluid: existence. Ain Shams Eng. J..

[29] Kumar RN, Gowda RP, Abusorrah AM, Mahrous YM, Abu-Hamdeh NH, Issakhov A, Prasannakumara BC (2021). Impact of magnetic dipole on ferromagnetic hybrid nanofluid flow over a stretching cylinder. Phys. Scr..

[30] Varun Kumar RS, Gunderi Dhananjaya P, Naveen Kumar R, Punith Gowda RJ, Prasannakumara BC (2022). Modeling and theoretical investigation on Casson nanofluid flow over a curved stretching surface with the influence of magnetic field and chemical reaction. Int. J. Comput. Methods Eng. Sci. Mech..

[31] Chu YM, Shankaralingappa BM, Gireesha BJ, Alzahrani F, Khan MI, Khan SU (2022). Combined impact of Cattaneo-Christov double diffusion and radiative heat flux on bio-convective flow of Maxwell liquid configured by a stretched nano-material surface. Appl. Math. Comput..

[32] Acharya N, Mabood F, Shahzad SA, Badruddin IA (2022). Hydrothermal variations of radiative nanofluid flow by the influence of nanoparticles diameter and nanolayer. Int. Commun. Heat Mass Transfer.

[33] Lv YP, Algehyne EA, Alshehri MG, Alzahrani E, Bilal M, Khan MA, Shuaib M (2021). Numerical approach towards gyrotactic microorganisms hybrid nanoliquid flow with the hall current and magnetic field over a spinning disk. Sci. Rep..

[34] Kodi R, Mopuri O, Sree S, Konduru V (2022). Investigation of MHD Casson fluid flow past a vertical porous plate under the influence of thermal diffusion and chemical reaction. Heat Transfer.

[35] Abdelhameed TN (2022). Entropy generation of MHD flow of sodium alginate (C6H9NAO7) fluid in thermal engineering. Sci. Rep..

[36] Ellahi R, Alamri SZ, Basit A, Majeed A (2018). Effects of MHD and slip on heat transfer boundary layer flow over a moving plate based on specific entropy generation. J. Taibah Univ. Sci..

[37] Khan M, Rasheed A, Salahuddin T (2020). Radiation and chemical reactive impact on tangent hyperbolic fluid flow having double stratification. AIP Adv..

[38] Bhatti MM, Arain MB, Zeeshan A, Ellahi R, Doranehgard MH (2022). Swimming of Gyrotactic microorganism in MHD Williamson nanofluid flow between rotating circular plates embedded in porous medium: application of thermal energy storage. J. Energy Storage.

[39] Ishtiaq F, Ellahi R, Bhatti MM, Alamri SZ (2022). Insight in thermally radiative cilia-driven flow of electrically conducting non-Newtonian Jeffrey fluid under the influence of induced magnetic field. Mathematics.

[40] Shehzad N, Zeeshan A, Shakeel M, Ellahi R, Sait SM (2022). Effects of magnetohydrodynamics flow on multilayer coatings of Newtonian and non-Newtonian fluids through porous inclined rotating channel. Coatings.

[41] Reddy SRR, Reddy PBA, Bhattacharyya K (2019). Effect of nonlinear thermal radiation on 3D magneto slip flow of Eyring-Powell nanofluid flow over a slendering sheet with binary chemical reaction and Arrhenius activation energy. Adv. Powder Technol..

[42] Shuaib M, Shah RA, Bilal M (2021). Von-Karman rotating flow in variable magnetic field with variable physical properties. Adv. Mech. Eng..

[43] Shuaib M, Shah RA, Durrani I, Bilal M (2020). Electrokinetic viscous rotating disk flow of Poisson-Nernst-Planck equation for ion transport. J. Mol. Liq..

[44] Bilal, M., Ayed, H., Saeed, A., Brahmia, A., Gul, T., & Kumam, P. (2022). The parametric computation of nonlinear convection magnetohydrodynamic nanofluid flow with internal heating across a fixed and spinning disk. *Waves Random Complex Media*, 1–16.

[45] Sun, T. C., DarAssi, M. H., Bilal, M., & Khan, M. A. (2022). The study of Darcy-Forchheimer hybrid nanofluid flow with the thermal slip and dissipation effect using parametric continuation approach over a rotating disk. *Waves in Random Complex Media*, 1–14.

[46] Ashraf W, Khan I, Andualem M (2022). Thermal transport investigation and shear drag at solid–liquid interface of modified permeable radiative-SRID subject to Darcy-Forchheimer fluid flow composed by γ-nanomaterial. Sci. Rep..

[47] Khader MM, Megahed AM (2013). Numerical solution for boundary layer flow due to a nonlinearly stretching sheet with variable thickness and slip velocity. Eur. Phys. J. Plus.

[48] Khan M, Rasheed A, Ali S, Azim QUA (2021). A novel formulation of 3D Jeffery fluid flow near an irregular permeable surface having chemical reactive species. Adv. Mech. Eng..

